# Household factors and gestational age predict diet quality of pregnant women

**DOI:** 10.1111/mcn.13145

**Published:** 2021-02-02

**Authors:** Mohammed Bukari, Mahama Saaka, Azaratu Masahudu, Zakari Ali, Abdul‐Latif Abubakari, Lillian Owusuwaa Danquah, Ayishetu Napari Abdulai, Abdul‐Razak Abizari

**Affiliations:** ^1^ Department of Nutritional Sciences, School of Allied Health Sciences University for Development Studies Tamale Ghana; ^2^ Medical Research Council Unit The Gambia at the London School of Hygiene and Tropical Medicine Banjul The Gambia

**Keywords:** diet quality, dietary diversity, food security, pregnancy, women

## Abstract

Adequate diet during pregnancy has positive effects on the mother and pregnancy outcome. Assessment of diet quality during pregnancy is particularly important in areas where household food security is suboptimal, to enable appropriate targeting and intervention. This study assessed diet quality and identified predicting factors among pregnant women in northern Ghana. A cross‐sectional study involving 403 pregnant women was conducted in May 2018. Pregnant women attending antenatal care clinics (ANC) were selected using simple random sampling technique. We assessed socio‐demographic characteristics, 24‐h recall and household food security. The minimum dietary diversity for women (MDD‐W) was used as a proxy measure for diet quality based on Food and Agricultural Organization (FAO) guidelines. Logistic regression models were fitted to determine the predictors of diet quality. The mean dietary diversity score (DDS) of 10 food groups was 4.4 ± 1.1 (95% CI: 4.3–4.5). Logistic regression showed that women of high educational level (adjusted odds ratio [AOR] = 2.42; 95% confidence interval [CI] [1.21–4.84]; *P* = 0.01), women of high household wealth index (AOR = 1.78; 95% CI [1.14–2.77]; *P* = 0.01], none/mild household hunger (AOR = 2.71; 95% CI [1.26–5.82]; *P* = 0.01), medium household size (6–15 members) (AOR = 1.66; 95% CI [1.04–2.66]; *P* = 0.03) and women of gestational age 20–35 weeks (AOR = 1.89; 95% CI [1.05–3.40]; *P* = 0.03) were more likely to have quality diets after adjusting for potential confounding variables. Diet quality among pregnant women was low and was predicted by educational level, household wealth, gestational age and food security. Women education and improvements in household food security could impact diets of pregnant women in northern Ghana.

**Key messages**

- Previous studies have focused on the effect of specific nutrients without taking account overall diet quality; this study assessed overall diet quality and its predictors among pregnant women.
- We found that diet quality among pregnant women was low and was predicted by gestational age, household food security, educational level and household wealth status.
- In this low‐income setting in West Africa, our findings could have important policy implications for improved female education, food security and antenatal care for improved diets during pregnancy and improve birth outcome.

## 1 INTRODUCTION

According to World Health Organization (WHO) (2012), pregnant women's diet should be balanced. Diet of pregnant women should be nutrient dense, as adequate nutrition during foetal development is important for long‐term physical health of the child. It is at this stage that adequate nutrient supply is critical for both the mother and her unborn child. Pregnancy is specifically a critical period for the programming of future populations (Danielewicz et al., 2017).

Maternal diet quality as measured by dietary diversity is crucial for both the mother and child. In particular, diet in the first trimester is important for the development and differentiation of various organs, whereas diet later in pregnancy may be important for overall foetal growth as well as brain development (Rifas‐Shiman et al., 2009). Inadequate nutrition, especially during the first trimester of pregnancy, may restrict foetal growth and has long‐term consequences on both the mother and child.

Maternal dietary diversity in Northern region of Ghana has been linked to low birth weight among children (Saaka, 2013). It has also been linked to nutrition sufficiency, micronutrient adequacy (Ali et al., 2014) and pregnancy outcome (Cheng et al., 2009; Gao et al., 2013; Shamim et al., 2016; Willy et al., 2016). As food insecurity is high among households in Northern region and has adverse implications on nutritional status (Saaka, 2016), diet quality is likely to be poor among them. Pregnant women may be worst affected as their nutrient needs are higher.

Iron deficiency anaemia is an important public health problem among pregnant women. During pregnancy, anaemia is harmful to both the woman and child, and it is associated with a higher risk of maternal and foetal morbidity and mortality (Kalaivani, 2009). Women need to have adequate storage of nutrients to meet the nutritional demands of pregnancy (Marangoni et al., 2016; World Health Organization & UNICEF, 2001). Assessments of diet quality among pregnant women in Ghana in most studies have focused on the effect of specific nutrients rather than overall diet quality. A single nutrient approach is a major limitation because multiple nutrient deficiencies are more likely to occur than single nutrient deficiencies in a low‐income setting like Ghana (Darnton‐Hill & Mkparu, 2015). Therefore, it is essential to shift from examining the effect of single nutrients to assessing the overall diet quality.

Assessing the determinants of diet quality during pregnancy is vital in ensuring optimal nutrition for both mother and the child (Madden, 2015). Thus, it is important to understand the factors associated with the intake of different food groups, particularly, intake of micronutrient‐rich foods by pregnant women who are most at risk of having diets with poor diversity. Such information may help policymakers and programme managers in designing interventions to address the problem of poor diet quality. Data on factors associated with inadequate dietary diversity among pregnant women in the Savelugu Nantong district in the northern region of Ghana are scarce. Therefore, this study assessed diet quality and its association with maternal factors including household wealth, food security, nutrition knowledge, education level, household size and gestational age in the district.

## 2 METHODS

### 2.1 Study area

The study was conducted in the Savelugu‐Nantong district in the northern region of Ghana; it has a single farming season, a potential for livestock and crop production (maize, millet, groundnut, cowpea, soybeans, yam, guinea corn and cassava) with a total population of 139,283 (Gss & Macro, 2009).

### 2.2 Study design, population and sampling

A cross‐sectional design was conducted in May 2018. The study population comprised pregnant women (aged 15–49 years) utilizing antenatal care in health facilities. Pregnant women at all stages of pregnancy were included in order to have a representative measure of diet quality among pregnant women.

We selected four health facilities for data collection (Savelugu hospital, Nantong clinic, Savelugu Reproductive and Child Health Centre [RCH] and Mbia Laboratory services.). The health facilities were selected using simple random sampling technique from a list of all health facilities in the district using Excel‐generated random numbers. We used the probability proportional to size (PPS) methodology to select 403 study participants from the four health facilities. Lists of pregnant women attending antenatal care clinics (ANC) in the four facilities were compiled to form the sampling frame.

Using the formula for estimating sample size of single proportions (Cochran, 2007) with a 95% confidence interval (CI), an assumed prevalence of 50% of pregnant women meeting minimum dietary diversity, a margin of error of 0.05 and a 5% contingency, the sample size was 403.

### 2.3 Measurement of study variables

The dependent variable was diet quality measured using a 24‐h dietary diversity score (DDS). The independent variables included the socio‐demographic characteristics of pregnant women such as age, highest education completed, occupation, religion, marital status, household size (grouped into 1–5, 6–15 and above 15 household members who live together and eat from a common cooking pot), household wealth and gestational age (weeks of pregnancy recorded from pregnant women's antenatal cards), household food security and nutrition knowledge. A brief description of the key study variables is as follows.

#### 2.3.1 Determination of household wealth status

Household wealth index was used as a proxy indicator for socio‐economic status (SES) of households. This was based on an earlier concept by Garenne and Hohmann‐Garenne (2003) and Saaka et al. (2017), using absolute measurement of household items such as housing quality, water availability and type of toilet facilities, and ownership of durable goods and livestock. Households were given scores depending on their possession of household assets. Household wealth index was the summation of individual scores of each household items; the scores ranged from 0 to 18. Households with wealth scores less than or equal to 13 were considered as having low wealth index score and households with wealth scores greater than 14 as high wealth index. A dichotomous variable had favourable model fit characteristics compared with the usual quantile system, which would have increased the number of parameters estimated for household wealth.

### 2.4 Measurement of diet quality

Diet quality was measured using DDS because it reflects intake of nutrient adequacy (Mirmiran et al., 2006). The Food and Agricultural Organization defines dietary diversity as the number of food groups an individual eats over the past 24 h (Kennedy et al., 2011). Dietary diversity of the pregnant women was assessed using the Food and Agricultural Organization (FAO) women dietary diversity questionnaire to determine women dietary diversity score (WDDS) (FAO, 2016). Emphasis was on dietary diversity; hence, food frequency or amount of food taken was not considered. The food groups that were included in the questionnaire were as follows: (1) grains, white roots and tubers and plantain (maize, rice, yam and wheat or foods made from these, e.g., bread, TZ, porridge and banku); (2) Vitamin A‐rich dark green leafy vegetables (Amaranth, okra leaves, baobab leaves, ayoyo, alefu and bra); (3) other Vitamin‐A rich vegetables (tomatoes, carrots, orange flesh potatoes, ripe mangoes, dawadawa pulp and watermelon); (4) other vegetables (garden eggs, okra fruits, garlic, green and red pepper and onion); (5) other fruits (orange, lemon, guava, ebony fruits, blackberry and banana); (6) flesh meat (beef, lamb, chicken and any organ meat, e.g., liver and offals); (7) eggs (chicken and guinea fowl); (8) nuts and seeds (groundnuts, cashew nuts, bungu [sesame] and melon seeds); (9) pulses (beans, peas and lentils); (10) milk and milk products (milk, cheese and yogurt). Respondents were given a score of one for consuming food from a specific food group and 0 for otherwise, this was used to calculate the total women dietary diversity score (WDDS) by summing scores from all foods eaten. The maximum women dietary diversity score (WDDS) was 10 for consuming food from all food groups and 0 for not consuming food from any food group at all. Respondents who had dietary diversity score of 5 and above were considered to have met the women dietary diversity (MDD‐W), because dietary diversity was used as a proxy for measuring diet quality, respondents who met their diet quality were considered to have quality diets.

### 2.5 Measurement of maternal nutrition knowledge

The knowledge questions were adapted from the guidelines for assessing nutrition‐related knowledge, attitudes and practices (KAP) manual published by FAO (Macías & Glasauer, 2014). The questionnaires were field tested and validated in several countries. The focus of our assessment was on basic maternal nutrition that is usually taught in nutrition education sessions to pregnant women. The content included food groups and their functions, balanced diet, nutrition during pregnancy and lactation, causes and prevention of anaemia, iron absorption enhancers and inhibitors and foods sources of key nutrients (iron and Vitamin A).

Maternal nutrition knowledge was measured on a continuous scoring scale. The assessment was based on nine questions on knowledge of nutrient rich foods required during pregnancy and on anaemia. A score of 1 was assigned when a correct answer was given otherwise 0 to give a maximum score of 9. Overall nutritional knowledge was rated by calculating the total of all the valid responses pregnant women made. The overall composite nutrition knowledge index ranged from a minimum of 2 to a maximum of 9. The median score was 6.0. The scores were divided into two categories: pregnant women whose overall nutrition knowledge score was less than the median score were considered to have low knowledge and those with scores of 6 and above as high knowledge.

### 2.6 Assessment of anthropometry and nutritional status

Measurement of mid‐upper arm circumference (MUAC) was used to assess nutritional status, as this measurement is independent of pregnancy and therefore it is an effective indicator for a woman's nutritional status throughout the reproductive life. MUAC was measured following WHO standard procedure (WHO, 1995). MUAC measurement was used to classify pregnant women as normal or malnourished; pregnant women with MUAC less 23 cm were considered malnourished, and those with MUAC greater than or equal to 23 cm were considered to be normal (Tang et al., 2016).

### 2.7 Measurement of household food security

Household food security was measured using a modified FANTA household food insecurity and access scale (HFIAS) (Coates et al., 2007). The HFIAS has previously been modified to simply reflect household hunger, the household hunger scale (HHS), using three questions and three sets of responses (Deitchler et al., 2011; Saaka et al., 2017; Silventoinen, 2003). The three responses (never, rarely or sometimes and often) were scored and used to classify food insecurity/hunger. Never was assigned a score of 0; rarely or sometimes had a score of 1; and often was assigned a score of 2. Therefore, 0 was the minimum score, and 6 was the maximum score. Scores of 0–1 indicate little to no household hunger, 2–3 indicate moderate household hunger, and 4–6 indicate severe household hunger (Deitchler et al., 2011). In regression analysis, we combined none and mild household hunger and those with medium or and severe as the second to reduce the possibility of data sparsity arising from empty cells in the fully adjusted model.

### 2.8 Quality assurance

Questionnaire was translated to Dagbanli and pretested on pregnant women attending antenatal care at health facilities. The questionnaires were administered by four trained undergraduate nutrition officers who attended a 3‐day training on administering the questionnaire and anthropometric assessment (MUAC). Data were collected at designated places at various health facilities to ensure privacy and were then checked on site for completeness on daily basis; incomplete questionnaires were identified, and respondents were followed up to complete missing data.

### 2.9 Data analysis

Data analysis was performed using SPSS version 21 (IBM Inc.). Means and standard deviations were used for continuous data. Frequencies and percentages were used for categorical data. Bivariate analyses were performed using chi‐square/Fisher exact test as preliminary analysis to identify predictors of women's dietary diversity. Variables showing evidence of association with the outcome variable from the preliminary analysis were included for multivariate logistic regression analysis. We used the variance inflation factor (VIF) to assess collinearity among variables in the regression model, variables did not show evidence of significant collinearity (VIF < 5) (Ringle et al., 2015). Independent factors such as gestational age, educational level, household wealth index and household size were accounted for in the multivariate logistic regression modelling.

### 2.10 Ethical statement

Ethical clearance for the study was obtained from the School of Allied Health Sciences, University for Development Studies, Tamale, Ghana. Informed consent was obtained from each participant before they enrolled on the study.

## 3 RESULTS

### 3.1 Socio‐demographic characteristics of study participants

The study involved 403 pregnant women with more than half (58.3%) of them between 21 and 30 years. The predominant religion in the setting was Islam (97.8%). Six out of every 10 respondents did not have formal education (61.5%), 96.8% were married and were mostly from the Dagomba ethnic group. Trading was the main occupation of the respondents (28.0%); however, 32.8% of were housewives. More than half (61.3%) of the pregnant women had high wealth index, and most (89.1%) of them did not experience food insecurity (Table 1).

**TABLE 1:** Socio‐demographic characteristics of study participants

Characteristic	Frequency	Percentage
Maternal age		
Below 20	63	15.6
21–30	235	58.3
30 above	105	26.1
Religion		
Islam	394	97.8
Christianity	9	2.2
Marital status		
Single	13	3.2
Married	390	96.8
Ethnicity		
Dagomba	381	94.5
Others	22	5.4
Mother's occupation		
Trader/vendor	113	28.0
Agricultural worker (e.g., farmer)	96	23.8
Office worker (civil servant)	4	1.0
Service worker (e.g., hairdresser and seamstress)	45	11.2
Educationist/researcher (e.g., teacher)	7	1.7
Health professional (e.g., nurse)	6	1.5
Nothing	132	32.8
Mother's highest level of education		
None	248	61.5
Low (primary and junior high)	107	26.6
High (at least senior high)	48	11.9
Gestational age (weeks)		
4–19	83	20.6
20–35	250	62.0
36–40	70	17.4
Wealth index		
High wealth	239	59.3
Low wealth	164	40.7
Household food insecurity		
None	359	89.1
Moderate	44	10.9
Nutritional status		
Normal	385	95.5
Underweight	18	4.5

### 3.2 Dietary diversity and frequency of consumption from specific food groups among pregnant women

The mean dietary diversity score (DDS) for 10 food groups at 95% CI (4.3–4.5) was 4.4 ± 1.1. Based on the women minimum dietary diversity (MDD‐W), 42.7% met the minimum dietary diversity of having consumed at least five food groups on the previous day.

The grains, roots and tubers food group was consumed from by all the pregnant women. Consumption of egg was poor with only 7.7% reporting consumption in the past 24 h prior to the survey (Table 2).

**TABLE 2:** Frequency of consumption from specific food groups among pregnant women

Food groups	Frequency	Percentage
Grains, white roots and tubers	403	100.0
Vitamin A‐rich dark vegetables	316	78.0
Other Vitamin A‐rich vegetables	14	3.5
Other vegetables	396	98.3
Other fruits	80	19.9
Flesh meat	92	22.8
Eggs	31	7.7
Nuts and seeds	250	62.0
Pulses (beans, peas and lentils)	112	27.8
Milk and milk products	86	21.3
Met MDD‐W	172	42.7

Abbreviation: MDD‐W, women minimum dietary diversity.

### 3.3 Nutritional knowledge of pregnant women

The proportion of pregnant women with high nutritional knowledge was 56.8%. Four out of every five pregnant women knew that breakfast was the most important meal and could mention at least one cause of anaemia. The proportion of pregnant women who knew that frequent suckling produces more breast milk was low (11.4%). Less than a half of the participants knew that coffee or tea could decrease iron (48%), although all of them knew that Vitamin C‐rich foods enhanced iron absorption. Most (82.4%) of pregnant women could mention at least three food sources of micronutrient in diets, and 72.0% of them knew the importance of feeding their children with fruits and vegetables. (Table 3).

**TABLE 3:** Maternal nutrition knowledge of pregnant women

Variable	Frequency	Percentage (%)
Proportion of mothers who knew that breakfast as meal is considered the most important for pregnant woman	324	80.4
Proportion of mothers that could mention at least 1 cause of anaemia	349	86.6
Proportion of mothers who knew that frequent sucklingproduces more breast milk	46	11.4
Proportion of mothers who knew when child has anaemia	300	74.4
Proportion of mothers who knew Vitamin‐C‐rich foods,such as fresh citrus fruits enhance iron absorption	403	100.0
Proportion of mothers who knew that coffee decreasesiron absorption when taken with meals	192	47.6
Proportion of mothers who knew that tea decreases ironabsorption when taken with meals	193	47.9
Proportion of mothers who could mention at least 3 food sources of micronutrients in their diet	332	82.4
Proportion of mothers who knew that fruits andvegetables are protective foods against diseases	290	72.0
Proportion of mothers having a high overall nutritional knowledge score index	229	56.8

### 3.4 Factors associated with MDD‐W

Logistic regression analysis showed that maternal educational level, household wealth index, household food security, household size (6–15 members) and gestational age were factors associated with having five or more food groups in the day (Table 4). Pregnant women who had high educational level (at least senior high school) were 2.4 times more likely to meet the MDD‐W (adjusted odds ratio [AOR] = 2.42; 95% CI [1.21–4.84]; *P* = 0.01) compared with those with lower educational attainment after adjusting for potential predictors included in the model. Pregnant women who lived in households with high wealth indices were also 1.8 times more likely to have higher diet quality (MDD‐W ≥ 5) (AOR = 1.78; 95% CI [1.14–2.77]; *P* = 0.01). Pregnant women of gestational ages between 20 and 35 weeks were more likely to meet the minimum dietary diversity (AOR = 1.89 CI [1.05–3.5]; *P* = 0.03) compared with those with older gestational ages (36–40 weeks). In comparison with participants who lived in households with moderate/severe food insecurity, those with none/mild food insecurity were 2.7 times more likely to meet the MDD‐W (AOR = 2.71; 95% CI [1.26–5.82]; *P* = 0.01). Pregnant women from small household size (6–15) were 1.66 times more likely of achieving minimum dietary diversity (AOR = 1.66; 95% CI [1.04–2.60]; *P* = 0.03), compared with those who lived in large households of above 15 members.

**TABLE 4:** Factors affecting maternal dietary diversity (≥5 MDD‐W): Logistic regression analysis

Predictor	AOR	95% CI for AOR		
		Lower	Upper	*P* value
Gestational age (reference: 36–40 weeks)				0.09
4–19 weeks	1.96	0.98	3.93	0.06
20–35 weeks	1.89	1.05	3.40	0.03
High household wealth index	1.78	1.14	2.77	0.01
Educational level (reference: None)				0.04
Low (primary and junior high)	1.31	0.81	2.12	0.27
High (at least senior high)	2.42	1.21	4.84	0.01
Household hunger scale (HHS) (none/mild)	2.71	1.26	5.82	0.01
Household size (reference: Above15)				0.09
1–5	1.09	0.59	2.00	0.79
6–15	1.66	1.04	2.66	0.03
Constant	0.09			<0.001

Abbreviations: AOR, adjusted odds ratio; CI, confidence interval; MDD‐W, women minimum dietary diversity.

Variables in the regression model did not show evidence of significant collinearity (VIF < 5). The set of variables accounted for 12.0% (*R* ^2^ = 0.12) of the variability in MDD‐W, an indication that other factors contribute to the dependent variable but were not measured in this study.

## 4 DISCUSSION

In the present study, we sought to assess diet quality of pregnant women and characterize the most important predictors of their diet. We found that high education level, high household wealth, food security, household size and women of young gestational (20–35 weeks) age were more likely to have quality diets.

In the current study, gestational age was associated to MDD‐W. As suggested in an earlier study, balanced nutrition during gestation has been identified as a prerequisite for a healthy pregnancy and birth outcomes (Bawadi et al., 2010). Nutrition knowledge among health personnel is often low (Kris‐Etherton et al., 2015; Mogre et al., 2018; Sunguya et al., 2013), and many trained nutritionists do not operate at this level of health care provision in Ghana. Therefore, as pregnancy progresses, it is likely difficult to give the appropriate diet counselling to motivate pregnant women to keep to a quality diet made of a variety of food groups. In addition, it is also possible that family food supplies and resources may be mobilized at this stage in preparation for delivery ceremonies such as naming ceremonies that are highly valued and tend to be food and resource intensive in this setting.

Similar to our study, previous studies (Amugsi et al., 2016; Shamim et al., 2016; Willy et al., 2017) reported that dietary diversity is influenced by education and is significantly high among pregnant women who have higher educational achievement. Also, Bodnar and Siega‐Riz (2002) confirmed that women who had better education consumed higher percentages of vegetables improving their diet quality. Another study by Ali et al. (2014) revealed otherwise. It is clear that increased educational level among our study group could have influenced dietary intake, because they were more likely to have understood regular nutrition education and made use of counselling services during ANC and other places.

According to Saaka (2016), food insecurity is highly prevalent in Northern Ghana. Arguably, food insecurity was low (10.9%) in this study. This could be because respondents from our study had higher purchasing power, accessibility and availability of food as compared with Saaka's study where respondents were from resource poor setting. Also, women especially pregnant women in the study area usually go through a process of socialization in their maiden pregnancies; often led by experienced mothers, they are taught how to combine staples to improve nutrition and sensitized on the need to eat regularly as pregnant women. Analysis of the type of food consumed from specific food groups by pregnant women in the past 24 h prior to the study revealed that there was association between household food insecurity and women dietary diversity. Ruel (2003) also revealed association between dietary diversity and food security. This shows how important dietary diversity and food security are; if there is dietary diversity, people could be food secured. Hence, it is essential to factor dietary needs of people into food security policies. This would help address food security crises while addressing nutritional needs of individuals. In addition, Harris‐Fry et al. (2015) and Hoddinott and Yohannes (2002) have also suggested that dietary diversity could measure food security; highlighting the need to give attention to both of them.

SES also plays a critical role in pregnancy outcome (Saaka, 2013). A significant association was found between SES and diet quality (Mbwana et al., 2016; Saaka, 2013). As SES increases, diet quality increases. Similar to this study, it has previously (Saaka, 2013) been stated that maternal dietary diversity was high in high socio‐economic class compared with women in low socio‐economic class. The association between household wealth index, household size and dietary diversity was confirmed by Shamim et al. (2016). In the current study, households with low size had better diet quality; this maybe because such households have lesser people to share food, because food is more culturally shared without necessarily considering pregnant women as a special group needing attention such as children. Thus, wealth and household size play critical role in determining dietary diversity. A study by Harris‐Fry et al. (2015) further highlights the role of wealth and literacy in improving dietary diversity.

Nutritional status during pregnancy is an important determinant of birth outcomes (Bodnar & Siega‐Riz, 2002). Our findings showed that 95.5% of participants were well nourished (normal), and no association was established between dietary diversity and nutritional status. Although in Kiboi's study Kiboi (2016), 19.3% were malnourished, there was a significant association between dietary diversity and nutritional status (Ali et al., 2014; Willy et al., 2016). The difference could be as a result of counselling services to pregnant women implemented as part of regular ANC service.

The *R* ^2^ value (12%) was rather small, indicating that all factors in the model together explain only 12% of the variability of the outcome. This may imply that more variables were not important or measured in the model. For instance, Abubakari et al. (2019) showed that food taboos are common during pregnancy and affect pregnancy outcome, which was not measured in the current study. Also, factors such as market availability of foods, intrahousehold distribution of foods and crop yields may not have been measured, hence the small *R* ^2^ value.

This study had some limitations. As diet quality was assessed using a recall method, we cannot rule out the potential for recall bias. If more educated women knew about the benefits of eating a diverse diet and therefore over recalled their consumption of specific foods to increase their diet diversity, our estimates on the relationship between high education and diet quality would be biased away from the null, implying that the real effect of higher education on diet quality is lower than reported. In addition, 24‐h recall might not usually be feasible as a result of participants not being entirely truthful or complying; nonetheless, they provide representative data of the population as a result of lesser burden on study participants relative to diet record method (Thompson & Subar, 2017). Even though women of all pregnancy stages were included in this study, the use of health facilities as target outlets for sampling pregnant women likely introduced some selection bias by gestational age where women of early pregnancy in particular were less likely to attend health facilities for antenatal care. This possibly underrepresented women of early gestational age in our sample.

Our findings could have important policy and further research implications. Policies that address household food security problems are likely to impact diet quality of pregnant women and possibly birth outcome. Encouraging girl child education can also have impacts on diets. Interestingly, the government of Ghana recently implemented a policy of free education for all school going age from basic level to the second cycle level with appropriate feeding interventions (Abizari et al., 2020). This study can provide supportive evidence to encourage girl child education in this part of Ghana where female education is low. Household wealth increase can also be improved to ensure improved diet quality pregnant women. Policies targeting poverty reduction could include provision of basic and durable household assets. Larger prospective studies should further investigate factors identified in this study and the effect of wider environmental exposures such as climate change on diet quality of pregnant women.

## 5 CONCLUSION

Diet quality among pregnant women was low and was predicted by educational level, household wealth, gestational age and food security. Women education and improvements in household food security could impact diets of pregnant women in northern Ghana.

## CONFLICT OF INTERESTS

The authors declare that they have no competing interest.

## CONTRIBUTIONS

MS conceptualized and supervised the study. MS, AM, ALA, LOD and ANA took part in the study design and data acquisition. MB, AM, ZA and MS took part in analysing and interpreting the data. MS, ZA and ARA reviewed it critically for important intellectual content. MB and AM wrote first draft of manuscript. MB was deeply involved in revising it for critical content. All authors read and approved the final manuscript.

6

## Data Availability

The data set supporting the findings of this study could be obtained from the corresponding author upon reasonable request.
